# Colon perforation caused by swallowing a shrimp leg: A case report

**DOI:** 10.1016/j.ijscr.2018.09.042

**Published:** 2018-10-04

**Authors:** En-Nung Kao, Kuo-Hsiu Liao, Teng-Wei Chen, De-Chuan Chan, Jyh-Cherng Yu

**Affiliations:** a Division of General Surgery, Department of Surgery, Taichung Armed Forces General Hospital, No.348, Sec.2, Chungshan Rd., Taiping Dist., Taichung City 41152, Taiwan; b Division of General Surgery, Department of Surgery, Tri-Services General Hospital, National Defense Medical Center, No.325, Sec.2, Chenggong Rd., Neihu District, Taipei City 114, Taiwan

**Keywords:** Colon, Perforation, Foreign body

## Abstract

**Introduction:**

Ingestion of a foreign body rarely results in perforation of the gastrointestinal tract. Foreign bodies such as dentures, fish bones, chicken bones, toothpicks and cocktail sticks have been known to cause bowel perforation; however, bowel perforation caused by shrimp leg has not been reported so far.

**Presentation of case:**

We report a case of a 69-years-old man who presented with a 4-months epigastric pain. Laboratory data revealed inflammation at the first hospital visit. Computed tomography revealed a hypodense lesion containing a hyperdense foreign body in the abdomen. Intra-abdominal abscess caused by foreign body perforation was diagnosed.

**Discussion:**

An increased incidence of perforation has been reported in association with Meckel’s diverticulum, the appendix, and diverticular disease. The abdominal abscess in this case was located beside the diverticulum of transverse colon, so the perforation most likely occurred in the diverticulum of transverse colon.

**Conclusion:**

Perforation of colon by shrimp leg is very rare and not reported currently. Although some cases have been successfully treated chronic foreign-body perforation with abscess by using antibiotics alone, typical treatments remains surgical drainage of the abscess and removal of foreign body.

## Highlights

• Colon perforation caused by swallowing a shrimp leg is the first reported case currently.
• Chronic perforation of colon may not need emergent surgery.
• Laparoscopic drainage and removal of shrimp leg is effect treatment to diminish hospital days.

## 1 Introduction

Report shows, most foreign bodies pass through the gastrointestinal tract without any consequence and perforation of the bowel by the foreign body occurs less than 1% [1]. Foreign bodies such as dentures, fish bones, chicken bones, toothpicks and cocktail sticks have been known to cause bowel perforation; however, bowel perforation caused by shrimp leg has not been reported so far. The most common sites of perforation are the narrowest parts of the bowel, such as the ileocecal valve or recto-sigmoid junction, but perforation of the transverse colon is rare [2]. We report an unusual case involving perforation of the transverse colon by a shrimp leg. This case has been reported in line with the SCARE criteria [14].

## 2 Case presentation

A 69-year-old man had epigastric pain after eating fried shrimp without peeling shell 4 months ago and the symptom subsided 3 days later from that date. However, he has had intermittent epigastric pain from then on. He called at our emergent department because of epigastric pain with fever up to 38.2 ℃ since 2 days ago. He denied constipation, diarrhea, but has nausea. He denied peptic ulcer history nor any systemic diseases. The epigastric pain will be alleviated by bending abdomen and aggravated by laying down. Physical examination showed epigastric tenderness without muscle guarding nor rebounding tenderness. Laboratory tests documented elevated inflammatory markers with C-reactive protein 7.6 mg/dL. Other laboratory data were within normal ranges.

**Fig. 1:**
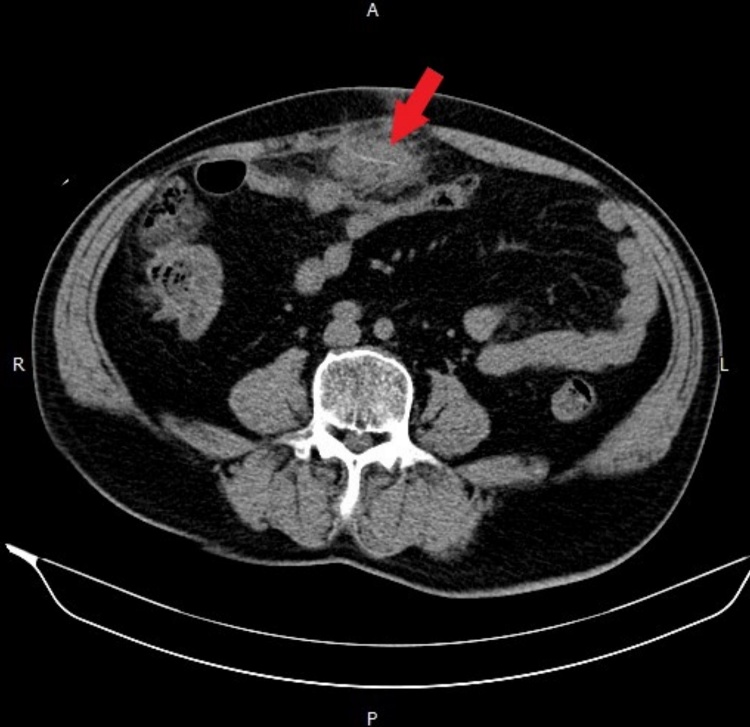
Intra-abdominal abscess with shrimp leg.

**Fig. 2:**
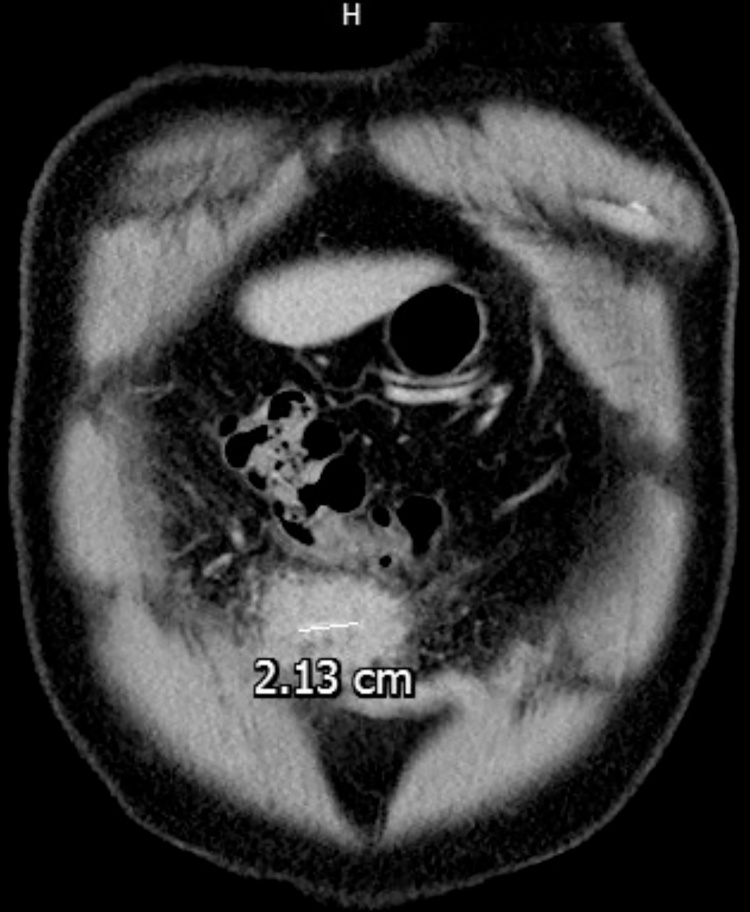
Intra-abdominal abscess near the transverse colon.

Computed tomography revealed a hypodense region 52 mm in diameter with a 21-mm hyperdense linear object beside the transverse colon. (Fig. 1, Fig. 2).

**Fig. 3:**
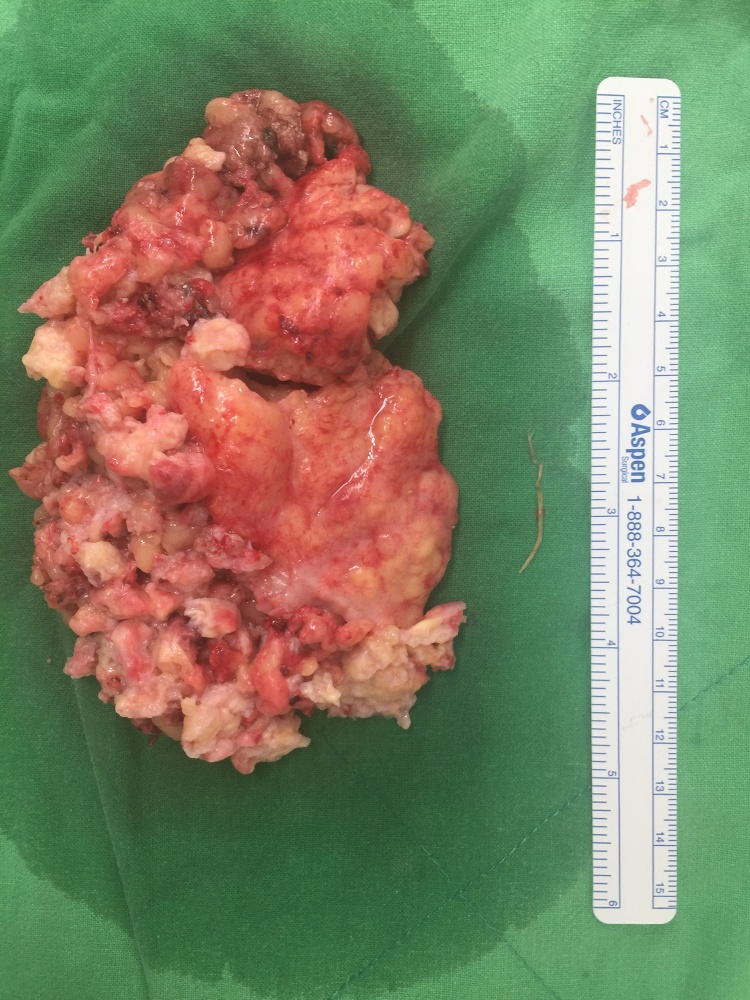
Shrimp leg in the abscess.

**Fig. 4:**
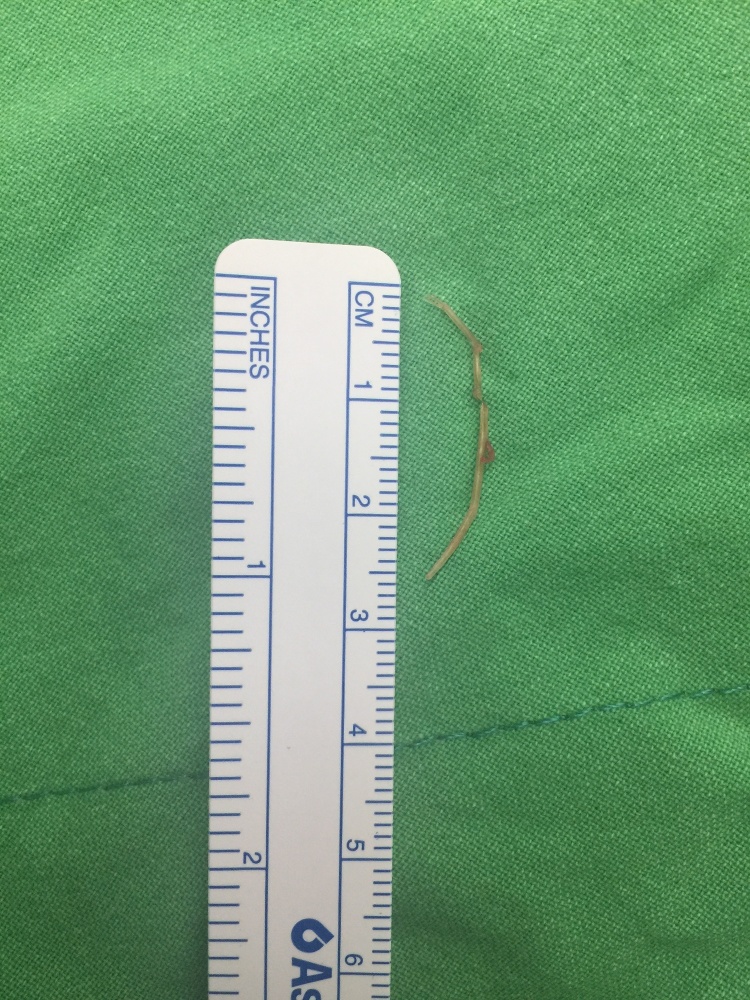
Shrimp leg is about 26-mm in length.

The patient was diagnosed as foreign body perforation of the transverse colon and intra-abdominal abscess. Although the patient described epigastric pain, he has remained able to eat for 4 months; he was treated with antibiotics (Sulbactam /Ampicillin, 6 g/d) at first. Persisted epigastric pain of the patient was still noted, so he asked for surgical intervention. Therefore, laparoscopic removal of the abscess and the foreign body with drainage was performed. During the operation, severe adhesion between abscess and diverticulum of T-colon was found. The foreign body was a 26-mm shrimp leg found in the abscess (Fig. 3, Fig. 4). The patient was discharged 3 days postoperatively with no complications.

## 3 Discussion

Bowel perforation from a foreign body is relatively rare and usually caused by fish or chicken bones, and toothpicks; [3] but perforation by shrimp leg is not reported currently. Common sites of perforation are the narrow parts of the bowel [2,3], while an increased incidence of perforation has been reported in association with Meckel’s diverticulum, the appendix, and diverticular disease [[8], [9], [10], [11], [12]]. The abdominal abscess in this case was located beside the diverticulum of transverse colon, so the perforation most likely occurred in the diverticulum of transverse colon. Although perforation of the small intestine is also possible, foreign body perforations of colon reportedly tend to present with a longer, more innocuous clinical presentation than perforations of the jejunum or ileum [3]. There are no evidence of narrow parts in the transverse colon. Therefore, we consider perforation of the diverticulum of transverse colon as the most likely scenario in this case.

In this case, the patient remembered eating fried shrimp without peeling shell 4 months earlier. The shrimp leg was narrow enough that the perforated site closed spontaneously, and allowing the patient to keep eating for 4 months without severe peritonitis. The foreign body is thus more like a shrimp leg according to its shape and the patient’s memory.

## 4 Conclusion

Perforation of colon by shrimp leg is very rare and not reported currently. Although some cases have been successfully treated chronic foreign-body perforation with abscess by using antibiotics alone [[4], [5], [6], [7]], typical treatments remains surgical drainage of the abscess and removal of foreign body. This case, the patient underwent laparoscopic drainage and removal of the foreign body, and showed a rapid recovery. Therefore, laparoscopic drainage of the abscess and removal of the foreign body can be an effective option for chronic cases.

## Conflicts of interest

There is no any conflicts of interest in this case report.

## Sources of funding

This research did not receive any specific grant from funding agencies in the public, commercial, or not-for-profit sectors.

## Ethical approval

Ethical approval has been exempted by Ethics Committee of Taichung Armed Forces General Hospital.

## Consent

Informed consent permit was signed by the patient for publication of this case report and associated images.

## Author contribution

En-Nung Kao – Primary and Corresponding author of the case report, contributed to surgical procedures, postoperative patient care, study conception and design, literature search, manuscript preparation and writing.

Kuo-Hsiu Liao – Secondary author, contributed to surgical procedures, postoperative patient care and manuscript editing and review.

Teng-Wei Chen – Contributed to postoperative patient care, and manuscript review.

De-Chuan Chan – Contributed to postoperative patient care, and manuscript review.

Jyh-Cherng Yu – Contributed to postoperative patient care, and manuscript review.

## Registration of research studies

Not applicable.

## Guarantor

En-Nung Kao.

Kuo-Hsiu Liao.
